# Surveillance ultrasonography for conservative treatment of femoral shaft fractures in young children

**DOI:** 10.1186/s13018-020-02149-9

**Published:** 2020-12-11

**Authors:** Hui Gao, Zhaoxia Wang, Yuxi Su

**Affiliations:** 1 Department of Ultrasound, Chongqing Key Laboratory of Pediatrics, Ministry of Education Key Laboratory of Child Development and Disorders; National Clinical Research Center for Child Health and Disorders; China International Science and Technology Cooperation base of Child Development and Critical Disorders, Children’s Hospital of Chongqing Medical University, Chongqing, People’s Republic of China; 2 Department II of Orthopedics; Chongqing Key Laboratory of Pediatrics, Ministry of Education Key Laboratory of Child Development and Disorders; National Clinical Research Center for Child Health and Disorders; China International Science and Technology Cooperation base of Child development and Critical Disorders; Children’s Hospital of Chongqing Medical University, Yuzhong District Zhongshan 2road 136#,, Chongqing, 400014 People’s Republic of China

**Keywords:** Femoral fractures, Radiation injuries, Ultrasonography, Doppler

## Abstract

**Background:**

The treatment for femoral shaft fracture (FSF) depends on the age of the patient. While the Pavlik harness is the first choice for patients under 6 months of age, spica casting is preferred for patients over 6 months and under preschool age. Minimally-invasive surgery using elastic stable intramedullary nails is also used in some cases. Skin traction is another treatment choice for some patients who are not candidates for the above methods. This study aimed to evaluate the feasibility of surveillance ultrasonography (US) for the conservative treatment of FSFs in young children.

**Materials and methods:**

This retrospective study included 92 children who were diagnosed with FSF in our hospital from April 2017 to May 2019. After applying the inclusion and exclusion criteria, they were divided into US surveillance (A) and control (B) groups. All patients received conservative treatment by skin traction. For group A, US was used to assess the femur fractures and adjust its reduction on days 1, 3, 5, 7, 10, and 14 until the fracture stabilized. For group B, the fractures were checked by radiographs on days 1, 3, 5, 7, 10, and 14 until the callus appeared. The FSF angle was measured using anteroposterior and lateral radiographs.

**Results:**

All patients were followed up for 18 months. The radiographic evaluation of both groups at the final follow-up showed a significant difference in the FSF angle. The radiograph times and accumulated radiation also showed significant differences between the two groups. However, there was no significant difference in the incidence of complications.

**Conclusions:**

For FSF closed reduction, surveillance US is a better option compared to radiographs in children treated by skin traction. This approach can significantly decrease exposure to X-ray radiation and improve the reduction.

**Level of evidence:**

III

## Introduction

Femoral shaft fracture (FSF) is one of the most common fractures in children, with an incidence rate of 20 per 100,000, accounting for 2% of all pediatric fractures [1]. The treatment for FSF depends on the age of the patient. The Pavlik harness is the first choice of treatment for patients under the age of 6 months [1, 2]. For patients over 6 months and younger than 6 years of age (preschool children), closed reduction and casting are widely accepted by most surgeons and patients due to their good outcomes. In recent years, an increasing number of surgeons have tended to choose elastic stable intramedullary nail (ESIN) as the fixation material because of the mini-invasive method, short hospitalization, short time in bed, and lower cost associated with it [1, 3–6]. However, some contraindications for ESIN in these children include multiple fractures and not isolated FSFs, excessive swelling in the thigh, and open fractures [7]. Complications associated with casting fixation include malunion of the fractures, such as angular or rotation deformity, skin compression, skin allergy caused by plaster, and the compartment syndrome, which cannot be diagnosed immediately and can lead to ischemic contracture [8, 9]. In our study, we focused on patients who were diagnosed with FSFs and underwent skin traction.

Ultrasonography (US) is widely used for the diagnosis of musculoskeletal injuries [10]. It has the advantages of no radiation, dynamic monitoring, and easy learning and usage, as well as low cost and easy availability of equipment [11–14]. Recently, an increasing number of surgeons have also found US to be useful in the diagnosis of fractures, especially in young children, wherein the bone is not ossified and covered by cartilage, such as the forearm [13], radial head [12], femur [15], and metatarsal [16] fractures. It has also been found to be useful in the diagnosis of occult or missed fractures in children [17]. In recent years, some studies have shown the feasibility of US in fracture reduction and even during surgery [12, 18]. We and others have previously demonstrated the usefulness of US in the guidance of radial neck fracture reduction during surgery [19, 20]. To the best of our knowledge, this is the first study on the use of surveillance US for the treatment of FSFs in children who were treated by skin traction.

## Materials and methods

This study was approved by the Children’s Ethics Committee of our hospital. All clinical records of patients in our hospital were reviewed from April 2017 to May 2019. The guardians or parents of the patients authorized us to use the clinical data of their children. The inclusion criteria were children who (a) were over 6 months and under 6 years of age, (b) had fresh femoral fractures, and (c) were treated with closed reduction and skin traction. Children over the age of 6 years with old femoral fractures, those who had undergone cast fixation, and those with no overlapping displacement were excluded from the study. The same physician analyzed all the clinical data used in the analysis. The radiographs including the anteroposterior and lateral radiographs, as well as the shaft angles, were measured accordingly. After analyzing the clinical data, the patients were divided into two groups according to the treatment method. The US surveillance group was defined as group A, and the control group was defined as group B. All patients received conservative treatment by skin traction. Group A comprised 29 patients (21 boys and 8 girls aged 29.9 ± 15.7 months), and group B comprised 63 patients (39 boys and 24 girls aged 30.3 ± 15.6 months). The clinical data, including hospitalization fees, dose of radiation, and exposure times, were recorded.

### Surveillance ultrasonography

**Fig. 1:**
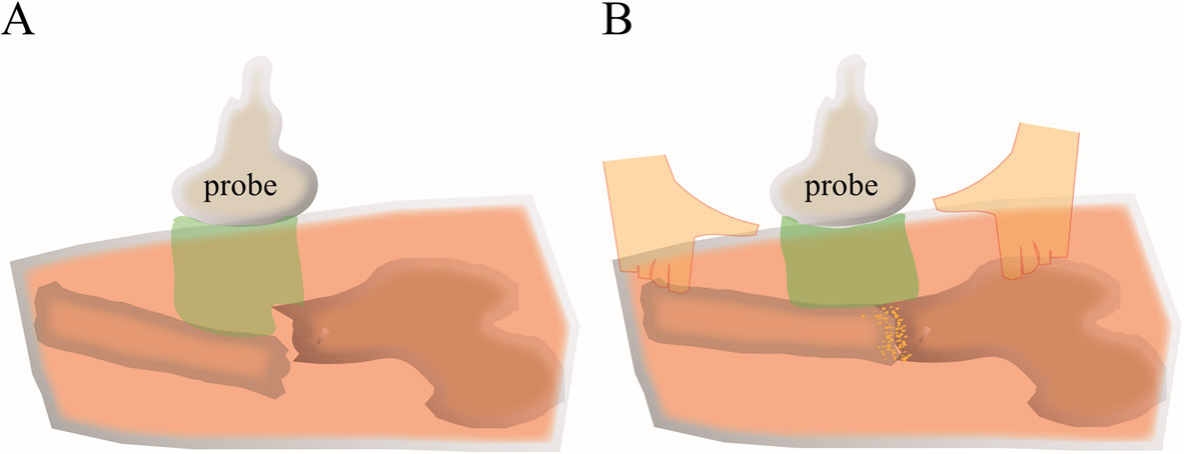
Closed reduction of FSF under surveillance US. **a** Checking the FSF using the ultrasound scanner. **b** Ultrasound surveillance and assistance for closed reduction of the FSF. FSF, femoral shaft fracture; US, ultrasonography

Once the patients were diagnosed with FSFs, they were carefully evaluated by the physicians for other combing fractures and injuries, including those caused by abuse. There was no need for anesthesia while performing skin traction and US surveillance. Skin traction was applied to all patients in group A after diagnosis. The hip and knee angles were kept at 30–45° of flexion and 30° of abduction. The leg was placed at 15° of external rotation to align the distal fragment with the external rotation of the proximal fragment to avoid rotation of the lower leg. With patients who could not be easily fixed in bed, a man-made feet brace was used to control the rotation of the lower legs. The traction weight was approximately 1/6–1/8 of the patient’s weight. Group B patients were monitored using bedside X-ray radiographs for alignment of the FSFs once or twice every week until the callus appeared. All group A patients were monitored using US every 1–3 days, especially during the first 2 weeks. As shown in Fig. 1, a mobile Philips real-time scanner with a 5-MHz linear probe and a hypoallergenic gel were used in this study. The fracture was observed as a break in the bone cortex, and the alignment angle was measured. The initial period of hematoma formation (1–2 days) was followed by the organization of the hematoma (approximately 1–2 weeks), and finally, the formation of the cartilage callus, which lasted approximately 3–12 weeks, depending on the patient’s ages and site of injury. Using US, the FSF was realigned by adjustment of the skin traction. Once the callus was visible in the X-ray radiographs, skin traction was stopped, and a man-made brace was applied for further fixation.

### Follow-up and evaluation

For group A, the femur fractures were checked by US, and the position was adjusted on the basis of the findings on days 1, 3, 5, 7, 10, and 14 until the fracture stabilized. For group B, the femur fractures were checked by radiographs on days 1, 3, 5, 7, 10, and 14 until the callus appeared. The FSF angle was measured on the basis of the anteroposterior and lateral radiographs.

### Statistical analyses

*T* tests were used to compare continuous variables, and *χ*^2^ tests were used for independent and categorical variables. Continuous variables are described as means plus standard deviation. Statistical analyses were performed using the SPSS software, version 13 (IBM, Armonk, NY). *P* < 0.05 was considered statistically significant.

## Results

A total of 92 patients were included in this study. Table 1 summarizes the demographic characteristics of the study participants. No significant differences were observed between the two groups in terms of age (*P* = 0.9130), sex (*P* = 0.3255), injured femoral side (*P* = 0.5767), mechanism of injury (*P* = 0.6201), and fracture types (distal, middle, and proximal) (*P* = 0.9973) (Table 1). Skin traction was applied to all patients based on the type of FSF. The duration of skin traction showed no significant difference between the two groups, indicating that US does not decrease skin traction time. An analysis of the complications of skin traction, such as skin problems, vessel injury, nerve injury, and acute compartment syndrome, revealed no significant differences between the two groups (Table 2).

**Table 1:** Demographic characteristics of patients

	Group A (with US)	Group B (without US)	*P* value^#^
Age			
Mean age (months)	29.9 ± 15.7	30.3 ± 15.6	0.9130
Gender			
Male	21	39	0.3255
Female	8	24	
Injured femur			
Left side	17	33	0.5767
Right side	12	30	
Injury mechanism			
Fall	11	25	0.6201
High fall	10	15	
Hit by a heavy object	2	9	
Pedestrian injury by vehicles	6	14	
FSF sites			
Distal	4	9	0.9973
Middle	20	43	
Proximal	5	11	

*US* Ultrasonography, *FSF* Femoral shaft fracture

^#^*P* < 0.05 was considered significant

**Table 2:** Evaluations and complications

	Group A (with US)	Group B (without US)	*P* value
Skin traction time (days)	15.8 ± 3.2	16.9 ± 5.5	0.8141
1st day angles on AP radiographs (degrees)			
< 10	9	13	0.6922
11–20	8	18	
21–30	7	21	
> 31	5	11	
1st day FSF angles on lateral radiographs (degrees)			
< 10	5	7	0.7940
11–20	10	23	
21–30	9	18	
> 31	5	15	
7th day angles on AP radiographs (degrees)			
< 10	15	12	0.0198^#^
11–20	9	26	
21–30	4	15	
> 31	1	7	
7th day FSF angles on lateral radiographs (degrees)			
< 10	9	9	0.1446^#^
11–20	13	27	
21–30	5	14	
> 31	2	13	
Final FSF angles on AP radiographs (degrees)			
< 10	19	16	0.0032^#^
11–20	8	29	
21–30	2	11	
> 31	0	5	
Final FSF angles on lateral radiographs (degrees)			
< 10	15	14	0.0316^#^
11–20	10	31	
21–30	3	9	
> 31	1	9	
X-radiographs times (*n*)	24.1 ± 4.5	7.2 ± 1.7	0.0001^#^
Radiation (mGy)	0.58 ± 0.11	0.18 ± 0.06	0.0001^#^
Cost (US dollar)	1087.1 ± 386.7	692.0 ± 435.3	0.0005^#^
Complications			
Skin problems	5	12	0.8357
Vessel injury	0	0	
Nerve injury	0	0	
Acute compartment syndrome	0	0	

*US* Ultrasonography, *FSF* Femoral shaft fracture, *AP* Anteroposterior

^#^*P* < 0.05 was considered significant

However, US significantly decreased the FSF angles not only on the anteroposterior (*P* = 0.0032) but also on the lateral (*P* = 0.0316) radiographs. It also significantly reduced the need for X-ray radiographs (*P* = 0.0001), and thereby, the exposure to radiation (*P* = 0.0001). Additionally, the patient’s hospitalization cost was significantly decreased in group A compared to group B (*P* = 0.0005).

## Discussion

In this study, we retrospectively evaluated the feasibility and benefits of surveillance US for the treatment of FSFs in patients undergoing skin traction. Our findings demonstrate that US can improve the overall outcomes and decrease the exposure to X-ray radiation in FSF treatment.

There is consensus regarding the treatment for FSFs in children of different ages. The Pavlik harness is the first choice of treatment for children under the age of 6 months. It has the advantages of not requiring anesthesia and causing less skin irritation and skin breakdown compared to an instant cast [8]. However, there is some dispute regarding the treatment in children between 6 months and 6 years of age. The spica cast is the first choice of treatment for children in this age group [1, 21]. In recent years, an increasing number of surgeons are opting for surgery [1, 3, 4].

However, there are some contraindications for spica casting. First, serious complications such as acute compartment syndrome, non-union, and skin problems can arise if the parents/guardians of the child are not careful. Second, the fracture must be an uncomplicated single femur fracture without any skin injury or fractures of other bones, such as the tibia or fibula. Third, there is an age limitation of 6 years. Fourth, general anesthesia is required [1]. In our study, all patients were ineligible for spica casting and were treated by skin traction. There were several reasons for opting for skin traction in these patients. First, for social reasons, most parents did not want sedation or anesthesia for their children since they worried that these drugs could affect the child’s intelligence. Kang et al. have reported that early postnatal exposure to isoflurane causes cognitive deficits [22]. Second, although most of the patients opted for the minimally-invasive ESIN, they had to be prepared for an open reduction in case closed reduction by ESIN insertion failed [1, 2]. Most physicians agree that conservative treatment achieves lower non-union rates compared to surgery. ESIN fixation is an invasive method with complications that include infection of the incision and bones, delayed union or non-union of the fractures, and the requirement of another operation to remove the ESIN. Third, it was usually the grandparents taking care of the patients since the parents were working, thus assuming responsibility for the children’s care. Fourth, consistent with the trend seen in developing countries, conservative treatments such as skin traction are significantly cheaper than surgery [7]. Fifth, due to the humid weather in our region, spica casting leads to several skin problems. Skin traction, together with a splint, is significantly more comfortable for most children. Lastly, abuse is very common in young children; thus, they are safer at the hospital, where it is possible to have more time to identify potentially abusive guardians [23].

**Fig. 2:**
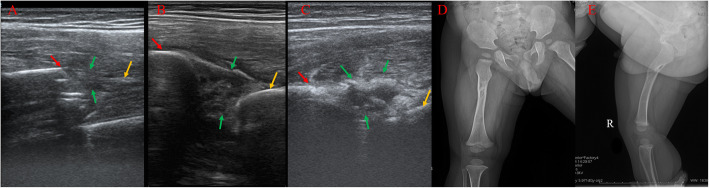
Images of a 2-year-old boy with FSF. The red and yellow arrows show the distal and proximal fractures, respectively. The green arrow shows the callus. **a** Ultrasound imaging on day 3 after injury, during the hematoma period. **b** Ultrasound imaging on day 8 after the injury, during the organization of the hematoma, which could be adjusted and reduced. This could not be visualized by radiographs (green arrow). **c** The appearance of the callus indicating the stabilization of the fracture, which can be seen on the radiographs. **d**, **e** Radiographs showing the anteroposterior and lateral views during the final follow-up

In line with several previous studies [12, 13, 15, 16], we also demonstrated the feasibility of surveillance US for the diagnosis and closed reduction of fractures in children. We have previously shown that US can improve the reduction of radial neck fractures in children [20]. Li et al. have reported that US can help in the reduction of lateral humerus super condylar fractures in children [24]. It has the advantage of not being static, as is the case with radiography, thereby dynamically monitoring the reduction. Additionally, it can also monitor the stability of the fractures. Once the callus appears in the X-ray radiographs, there is limited chance for further reduction of the fractures. However, US allows clear visualization of the initial formation of the hematoma (1–3 days) followed by its organization (1–2 weeks). During these periods, fractures that have not stabilized can easily be adjusted on the basis of the US findings (Fig. 2).

Newborns and infants are notably more susceptible to the harmful effects of X-ray radiation. However, X-ray or CT are still widely used in these patients [25]. The Alliance for Radiation Safety in Pediatric Imaging emphasized through their Gently Campaign the need to decrease exposure to radiation in young children. At our clinical center, we also emphasize the ALARA (as low as reasonably achievable) principle [26], by which we protect not only the patients and guardians, but also our staff. Considering that the accumulation of X-ray radiation may lead to cancers such as thyroid carcinomas and leukemia, we try our best to protect the patients and staff. In our study, surveillance US was found to significantly decrease the exposure to X-ray radiation. We also enrolled a US department physician to monitor fracture reduction in our study.

There are some limitations to our study. First, this was a retrospective study. To rule out any existing biases, a prospective randomized study may be needed. Second, our study had a small sample size. To confirm and validate the findings, a larger study or a multicenter collaboration may be needed. Third, a US physician and bedside machine were needed for this study. In future studies, physicians and pediatric orthopedic surgeons should work in close cooperation for closed reduction and adjustment of fractures. Fourth, orthopedic surgeons and US physicians should be trained, and the learning curve should be further studied to determine if this method is widely applicable.

## Conclusions

Although spica casting and ESIN are commonly used for the treatment of FSFs in preschool children, skin traction is still widely used in some areas with good outcomes. In this study, we demonstrated that US guidance for monitoring FSFs is feasible in children undergoing skin traction. It could significantly reduce the exposure to X-ray radiation during the traction period. Although there are some limitations, orthopedic surgeons should be encouraged to use US in accordance with the ALARA principle.

## Data Availability

Please contact the authors for data requests.

## Abbreviations

FSF: Femoral shaft fracture
ESIN: Elastic stable intramedullary nailing
US: Ultrasonography
ALARA: As low as reasonably achievable
